# Febrile neutropenia (FN) occurrence outside of clinical trials: occurrence and predictive factors in adult patients treated with chemotherapy and an expected moderate FN risk. Rationale and design of a real-world prospective, observational, multinational study

**DOI:** 10.1186/s12885-018-4838-z

**Published:** 2018-09-24

**Authors:** Bernardo Leon Rapoport, Matti Aapro, Marianne Paesmans, Ronwyn van Eeden, Teresa Smit, Andriy Krendyukov, Jean Klastersky

**Affiliations:** 1 The Medical Oncology Centre of Rosebank, 129 Oxford Road, Saxonwold, Johannesburg, 2196 South Africa; 2 Department of Immunology, Faculty of Health Sciences, University of Pretoria, Pretoria, South Africa; 3 Institut Multidisciplinaire d’Oncologie, Clinique de Genolier, Genolier, Switzerland; 4 Information Management Unit, Institut Jules Bordet, Brussels, Belgium; 5 Hexal AG, Holzkirchen, Germany; 6 Medical Oncology, Institut Jules Bordet, Brussels, Belgium

**Keywords:** Chemotherapy-induced neutropenia, Febrile neutropenia, Neutropenia, Chemotherapy, Cytotoxic chemotherapy, Myelosuppressive chemotherapy

## Abstract

**Background:**

Febrile neutropenia (FN) is a common occurrence during chemotherapy. Granulocyte colony-stimulating factors (G-CSFs) can significantly reduce the risk of FN. International guidelines recommend G-CSF for patients receiving chemotherapy with FN risk of ≥20% or 10% to 20% with defined risk factors. Prophylaxis is not typically recommended for FN risk of < 10%; however, few studies have investigated FN incidence in lower-risk patients in real-world settings and tried to identify higher-risk subgroups.

**Methods:**

This real-world prospective, observational, multinational study aims to estimate the rate of development of FN with a chemotherapy line expected to be associated with a 10% to 20% risk of FN. Eligible patients (> 18 years of age) will have a solid tumour or Hodgkin/non-Hodgkin lymphoma and a planned chemotherapy regimen with expected risk of FN of 10% to 20% (according to published guidelines). Patients will be observed for the duration of the chemotherapy line (first cycle administered without FN prophylaxis). Primary endpoint is incidence of FN after the first chemotherapy cycle. Secondary outcomes include: FN-associated morbidity and mortality; time to first FN occurrence; other FN risk factors and impact of FN on quality of life. A risk model using occurrence of FN as a binary outcome will be developed. Data will be stratified by age, comorbidities and other risk factors.

**Discussion:**

This study will provide insight into the real FN risk for common chemotherapy regimens and predictive factors for FN, including patients generally excluded from randomised clinical trials, from which reported FN rates have been variable. This study builds on knowledge of predictive factors from other research and will provide information on patients with 10% to 20% FN risk.

## Background

Neutropenia is a major cause of infection-related morbidity and mortality in patients treated with myelosuppressive chemotherapy regimens [1, 2]. Febrile neutropenia (FN) is the most serious manifestation of neutropenia and a key driver of chemotherapy dose delays and/or reductions, which may impact treatment efficacy [2, 3]. The development of FN often leads to increased treatment costs and longer hospital stays, and may also be associated with reduced quality of life (QoL) [1].

FN occurs frequently during chemotherapy. In a retrospective cohort study, FN occurred in 13% to 21% of patients receiving common myelosuppressive chemotherapy regimens for metastatic solid tumours, most frequently during the first cycle (23% to 36%) [4]. Granulocyte colony-stimulating factors (G-CSFs) can be used prophylactically to reduce the risk, severity and duration of FN, and as an adjunct to support the delivery of dose-dense (increased frequency) or dose-intense (increased dose) myelosuppressive regimens [2, 5]. The use of G-CSFs as primary prophylaxis for the prevention of FN has been shown to reduce the relative risks of FN by 46% on average across 15 studies in a systematic review (> 90% in a study of pegfilgrastim), infection-related mortality by 45% and early deaths by 40% [6]. In a study of G-CSF (filgrastim) in patients with early breast cancer treated with epirubicin-cyclophosphamide chemotherapy, the rate of FN was 1% in the G-CSF arms compared with 7% in the control arms (*P* = .004) [7].

Current evidence-based guidelines from the European Society for Medical Oncology (ESMO), American Society of Clinical Oncology (ASCO), and National Comprehensive Cancer Network (NCCN) recommend prophylaxis with G-CSF for patients treated with chemotherapy with an FN risk ≥20%, and for patients receiving chemotherapy with an FN risk of 10% to 20% if they also present with risk factors including age ≥ 65 years, poor performance status and prior FN [2, 5, 8]. Prophylaxis is not recommended for most patients receiving chemotherapy with an FN risk < 10% [2, 5, 8]; however, in absolute numbers, many patients with an estimated risk of < 20% develop FN with rates of complications similar to those of patients with a high risk of FN development [9]. There is evidence to suggest that patients at lower risk of FN may respond better to G-CSF and that reduced doses of G-CSF might be effective in these patients [8], which would make prophylaxis more cost-effective. In the systematic review of 15 studies, FN risk was reduced with G-CSF, compared with controls across baseline risks from 17 to 78% of FN in control patients, although the trend for greater benefits of G-CSF at lower FN risk levels was no longer significant when the pegfilgrastim or the two studies with baseline risk around 20% were excluded [6]. Supporting data comes mainly from clinical trials and few studies have investigated the occurrence of FN in lower risk patients in real-life settings. Moreover, high-risk sub-populations, such as the elderly or those with comorbidities, are often underrepresented in randomised controlled trials (RCTs), despite convincing evidence that older age and comorbidities increase the risk of developing FN during chemotherapy [10–12].

## Methods

### Aim

The primary objective of this real-world study is to estimate the rate of patients who will develop at least one episode of FN when treated with a chemotherapy regimen (new chemotherapy line) expected to be associated with a moderate (10% to 20%) risk of FN, according to published guidelines.

### Study design

This is a prospective, observational, multinational, multicenter study which began recruiting patients in December 2016. Study sites are currently in Belgium, India and South Africa. A total of 20 sites worldwide are expected to participate in the study, with at least five eligible patients enrolling, on average, at each site every month. The accrual period is expected to be up to 12 months in duration once the total number of sites is reached, with the study end at 9 months after the end of this period.

Patients are registered through a secure website as soon as they have signed informed consent forms and before the start of chemotherapy. Patients will be observed for the duration of the chemotherapy line (up to 6 cycles and up to 30 days after the last administration of chemotherapy).

### Study endpoints

The primary outcome measure is incidence of FN (i.e. absolute neutrophil count (ANC) < 0.5 IU and temperature ≥ 38.5 °C) after the first cycle of a chemotherapy line (in adult patients treated with a regimen expected to be associated with a moderate risk of FN, i.e. 10% to 20%).

Secondary outcome measures include overall incidence of FN after all chemotherapy cycles, incidence of complicated FN after each chemotherapy cycle, all cycles rates of morbidities (diarrhea or oral mucositis, using Common Toxicity Criteria 4.0) that might increase the risk of infectious complications, and mortality (before resolution of the febrile neutropenic episode, i.e. ANC > 0.5 IU and temperature < 38 °C for a period of 5 consecutive days), time to first occurrence of FN, distribution of cycle number for the first episode of FN, impact of risk of neutropenia (graded according to Common Toxicity Criteria 4.0), and FN on QoL (QoL will be assessed at selected participating sites only using the Functional Assessment of Cancer Therapy-Neutropenia [FACT-N]), other risk factors for FN beyond the chemotherapy regimen, and validation of the Bozcuk Score for FN occurrence [13].

### Inclusion criteria

Patients aged over 18 years with a diagnosis of a solid tumour or Hodgkin/non-Hodgkin lymphoma, with planned administration of a chemotherapy line from a regimen on the list of acceptable regimens (Table 1), to be started during the study accrual period (any line of chemotherapy; adjuvant/neo-adjuvant or metastatic setting); no planned administration of growth factors; no previous inclusion in the study for another chemotherapy line; provision of written (unless national law permits non-written), independent ethics committee-approved informed consent to participate in the study; willingness to fill in QoL questionnaires on days 1 and 8 of the first chemotherapy cycle, as well as compliance for blood sampling on the same days (selected study centers only, in which participation will be mandatory).

**Table 1:** List of acceptable chemotherapy regimens

Cancer type	Regimen and Dosing
Bladder cancer	**M-VAC** Interval between cycles: 4 weeks Cisplatin: 70 mg/m^2^ Doxorubicin: 30 mg/m^2^ Vinblastine: 3 mg/m^2^ Methotrexate: 90 mg/m^2^
Breast cancer	**AC** Interval between cycles: 3 weeks Cyclophosphamide: 600 mg/m^2^ Doxorubicin: 60 mg/m^2^ **AC → P (sequential regimen) for the P (paclitaxel) cycles** Interval between cycles: 3 weeks Paclitaxel: 175 mg/m^2^ **AC → T (sequential regimen) for the T (docetaxel) cycles** Variant 1: AC → T Interval between cycles: 4 weeks Docetaxel: 100 mg/m^2^ Variant 2: AC → T Interval between cycles: 1 week Docetaxel: 35 mg/m^2^ **AV** Interval between cycles: 3 weeks Doxorubicin: 50 mg/m^2^ Vinolrebine: 50 mg/m^2^ **CAPE-T** Interval between cycles: 3 weeks Capecitabine: 35000 mg/m^2^ Docetaxel: 75 mg/m^2^ **CMF** Interval between cycles: 4 weeks Cyclophosphamide: 1400 mg/m^2^ Fluorouracil: 1200 mg/m^2^ Methotrexate: 80 mg/m^2^ **EC** Interval between cycles: 3 weeks Cyclophosphamide: 600 mg/m^2^ Epidoxorubicin: 100 mg/m^2^ **FEC → T (sequential regimen) for the T (docetaxel) cycles only** Interval between cycles: 3 weeks Docetaxel: 100 mg/m^2^ **FEC** Interval between cycles: 4 weeks Cyclophosphamide: 1050 mg/m^2^ Epirubicin: 120 mg/m^2^ Fluorouracil: 1000 mg/m^2^ **P** Interval between cycles: 3 weeks Paclitaxel: 250 mg/m^2^ **EC → T (sequential regimen) for the T (docetaxel) cycles** Interval between cycles: 3 weeks Docetaxel: 100 mg/m^2^ **T** Interval between cycles: 3 weeks Docetaxel: 100 mg/m^2^ **TC** Interval between cycles: 3 weeks Cyclophosphamide: 600 mg/m^2^ Docetaxel: 75 mg/m^2^
Cervical cancer	**CDDP/PACLI** Interval between cycles: 3 weeks Cisplatin: 50─75 mg/m^2^ Paclitaxel: 135 mg/m^2^ **CDDP/TOPOTECAN** Interval between cycles: 3 weeks Cisplatin: 50 mg/m^2^ Topotecan: 2.25 mg/m^2^ **IRINOTECAN** Interval between cycles: 6 weeks Irinotecan: 500 mg/m^2^ **TOPOTECAN** Interval between cycles: 4 weeks Topotecan: 7.5 mg/m^2^
Colorectal cancer	**5FU/LEUCO** Interval between cycles: 4 weeks Fluorouracil: 2125 mg/m^2^ Leucovorin: 100 mg/m^2^ **FOLFIRI** Variant 1: FOLFIRI Interval between cycles: 2 weeks Fluorouracil: 2000─2400 mg/m^2^ Irinotecan: 180 mg/m^2^ Leucovorin: 200─400 mg/m^2^ Variant 2: FOLFIRI Interval between cycles: 1 week Fluorouracil: 2300 mg/m^2^ Irinotecan: 80 mg/m^2^ Variant 3: FOLFIRI Interval between cycles: 2 weeks Fluorouracil: 2000 mg/m^2^ Irinotecan: 180 mg/m^2^ **FOLFOX** Interval between cycles: 2 weeks Fluorouracil: 1600 mg/m^2^ Leucovorin: 200 mg/m^2^ Oxaliplatin: 85 mg/m^2^
Gastric cancer	**CDDP/IRINO** Interval between cycles: 3 weeks Cisplatin: 60 mg/m^2^ Irinotecan: 130 mg/m^2^ **DOCE/IRINO** Interval between cycles: 3 weeks Docetaxel: 30 mg/m^2^ Irinotecan: 140 mg/m^2^ **ECF** Interval between cycles: 3 weeks Epirubicin: 50 mg/m^2^ Cisplatin: 60 mg/m^2^ Fluorouracil: 200 mg/m^2^ **ECX** Interval between cycles: 3 weeks Epirubicin: 50 mg/m^2^ Cisplatin: 60 mg/m^2^ Capecitabin: 26250 mg/m^2^ **EOF** Interval between cycles: 3 weeks Epirubicin: 50 mg/m^2^ Oxaliplatin: 130 mg/m^2^ Fluorouracil: 200 mg/m^2^ **EOX** Interval between cycles: 3 weeks Epirubicin: 50 mg/m^2^ Oxaliplatin: 130 mg/m^2^ Capecitabine: 26250 mg/m^2^ **FOLFOX** Interval between cycles: 2 weeks Fluorouracil: 3400 mg/m^2^ Oxaliplatin: 100 mg/m^2^
Germ cell tumors	**BEP followed by EP** Interval between cycles: 3 weeks Bleomycin: 90 U Cisplatin: 100 mg/m^2^ Etoposide: 500 mg/m^2^ **CDDP/VP16** Interval between cycles: 3 weeks Cisplatin: 100 mg/m^2^ Etoposide: 300─500 mg/m^2^
Hodgkin lymphoma	**Stanford V** Interval between cycles: 4 weeks Bleomycin: 10 U Doxorubicin: 50 mg/m^2^ Etoposide: 120 mg/m^2^ Mechlorethamine: 6 mg/m^2^ Vinblastine: 12 mg/m^2^ Vincristine: 2.8 mg/m^2^
Non-Hodgkin lymphoma	**ACOD** Interval between cycles: 3 weeks Cyclophosphamide: 1000 mg/m^2^ Doxorubicin: 50 mg/m^2^ Vincristine: 2.4 mg/m^2^ **CCOP** Interval between cycles: 4 weeks Cyclophosphamide: 750 mg/m^2^ Liposomal Doxorubicin: 30 mg/m^2^ Vincristine: 2 mg/m^2^ **CHOP** Interval between cycles: 2 or 3 weeks Cyclophosphamide: 750–1200 mg/m^2^ Doxorubicin: 50–75 mg/m^2^ Vincristine: 1.4 mg/m^2^ **DA-EPOCH** Interval between cycles: 3 weeks Cyclophosphamide: 3750 mg/m^2^ Doxorubicin: 40 mg/m^2^ Etoposide: 200 mg/m^2^ Vincristine: 1.6 mg/m^2^ **GMOX-R (with rituximab)** Interval between cycles: 2 or 3 weeks Gemcitabine: 1000 mg/m^2^ Oxaliplatine: 100 mg/m^2^ **GC** Variant 1: Interval between cycles: 4 weeks Cisplatin: 100 mg/m^2^ Gemcitabine: 3000 mg/m^2^ Variant 2: Interval between cycles: 3 weeks Cisplatin: 75 mg/m^2^ Gemcitabine: 2000 mg/m^2^ **MEGACHOP (with rituximab)** Interval between cycles: 3 weeks Cyclophosphamide: 1200 mg/m^2^ Doxorubicin: 75 mg/m^2^ Vincristine: 2 mg/m^2^ **RFM (with rituximab)** Interval between cycles: 4 weeks Fludarabine: 75 mg/m^2^ Mitoxantrone: 10 mg/m^2^
Non-small cell lung cancer	**Carbo/PACLI** Interval between cycles: 3 weeks Carboplatin: 6 AUC Paclitaxel: 200 mg/m^2^ **CDDP/DOCE** Interval between cycles: 3 weeks Cisplatin: 75 mg/m^2^ Docetaxel: 75 mg/m^2^ **CDDP/PACLI** Interval between cycles: 3 weeks Cisplatin: 50─75 mg/m^2^ Paclitaxel: 135 mg/m^2^ **CDDP/VNR** Variant 1: CDDP/VNR Interval between cycles: 4 weeks Cisplatin: 100 mg/m^2^ Vinorelbine: 100 mg/m^2^ Variant 2: CDDP/VNR Interval between cycles: 4 weeks Cisplatin: 100 mg/m^2^ Vinorelbine: 120 mg/m^2^ **CDDP/VP16** Interval between cycles: 3 weeks Cisplatin: 100 mg/m^2^ Etoposide: 300─500 mg/m^2^ **VIG** Variant 1: VIG Interval between cycles: 3 weeks Gemcitabine: 1800 mg/m^2^ Ifosfamide: 3000 mg/m^2^ Navelbine: 45 mg/m^2^ Variant 2: VIG Interval between cycles: 3 weeks Gemcitabine: 2000 mg/m^2^ Ifosfamide: 3000 mg/m^2^ Navelbine: 50 mg/m^2^ **T** Interval between cycles: 3 weeks Docetaxel: 75 mg/m^2^
Occult primary adenocarcinoma	**DOCE/GEMCI** Interval between cycles: 3 weeks Docetaxel: 75 mg/m^2^ Gemcitabine: 2000 mg/m^2^
Esophageal cancer	**CDDP/IRINO** Interval between cycles: 3 weeks Cisplatin: 60 mg/m^2^ Irinotecan: 130 mg/m^2^ **ECF** Interval between cycles: 3 weeks Epirubicin: 50 mg/m^2^ Cisplatin: 60 mg/m^2^ Fluorouracil: 200 mg/m^2^ **ECX** Interval between cycles: 3 weeks Epirubicin: 50 mg/m^2^ Cisplatin: 60 mg/m^2^ Capecitabin: 26250 mg/m^2^ **EOF** Interval between cycles: 3 weeks Epirubicin: 50 mg/m^2^ Oxaliplatin: 130 mg/m^2^ Fluorouracil: 200 mg/m^2^ **EOX** Interval between cycles: 3 weeks Epirubicin: 50 mg/m^2^ Oxaliplatin: 130 mg/m^2^ Capecitabine: 26250 mg/m^2^
Ovarian cancer	**CARBO/DOCE** Interval between cycles: 3 weeks Carboplatin: 5 AUC Docetaxel: 75 mg/m^2^ **CARBO/PLACLI → TOPOTECAN (sequential treatment) only for the topotecan cycles** Interval between cycles: 3 weeks Topotecan: 7.5 mg/m^2^ **TOPOTECAN** Interval between cycles: 3 weeks Topotecan: 7.5 mg/m^2^
Pancreatic cancer	**GEMCI/IRINO** Interval between cycles: 3 weeks Gemcitabine: 2000 mg/m^2^ Irinotecan: 300 mg/m^2^
Prostate cancer	**CABAZITAXEL** Interval between cycles: 3 weeks Cabazitaxel: 25 mg/m^2^ **T** Interval between cycles: 3 weeks Docetaxel: 75─100 mg/m^2^
Small-cell lung cancer	**CARBO/VP16** Interval between cycles: 3 weeks Carboplatin: 300 mg/m^2^ Etoposide: 900 mg/m^2^ **CAV** Interval between cycles: 3 weeks Cyclophosphamide: 750 mg/m^2^ Doxorubicin: 40 mg/m^2^ Vincristine: 1.3 mg/m^2^ **CDE** Interval between cycles: 3 weeks Cyclophosphamide: 1000 mg/m^2^ Doxorubicin: 45 mg/m^2^ Etoposide: 300 mg/m^2^ **CDDP/VP16** Interval between cycles: 3 weeks Cisplatin: 100 mg/m^2^ Etoposide: 300─500 mg/m^2^
Soft tissue sarcoma	**T** Interval between cycles: 3 weeks Docetaxel: 100 mg/m^2^
Uterine cancer	**T** Interval between cycles: 3 weeks Docetaxel: 100 mg/m^2^

The list of acceptable regimens with expected risk of FN in the range of 10% to 20% was compiled according to published guidelines. Other regimens may be added to the list if an investigator has documented evidence of expected FN risk in this range. The updated list of chemotherapy regimens is available online.

### Exclusion criteria

Patients scheduled to receive a chemotherapy regimen not on the list of acceptable regimens; receiving FN primary prophylaxis with antibiotics or any available G-CSF; prior treatment with high-dose chemotherapy and/or stem cell transplantation; abnormal kidney (creatinine > 1.5× upper limit of normal) and liver function (aspartate transaminase and alanine transaminase > 2× upper limit of normal).

### List of acceptable chemotherapy regimens

Before starting recruitment, investigators at any participating site may submit a chemotherapy regimen not present on the list to the study coordinating committee, accompanied by convincing data, to demonstrate that this regimen has an expected moderate risk of FN without administration of growth factors. If a proposed regimen is accepted by the committee, it will be added to the list.

### Investigations

Investigations will include assessment of eligibility, patient registration, and allocation of study number through a secured website.

On day 1 of the first chemotherapy cycle before chemotherapy, a routine blood sample with haematological counts is taken, a QoL questionnaire (FACT-N) is distributed to the patient (optional), and checks for verifying correct completion by the patient (selected sites only) and an assessment of Charlson comorbidity score are carried out. At selected sites, on day 8 of the first chemotherapy cycle, a blood sample with haematological counts is taken (optional), a QoL questionnaire (FACT-N) is distributed to the patient (optional), and checks for verifying correct completion by the patient are carried out. On day 1 of subsequent chemotherapy cycles, routine blood samples with haematological counts are taken.

### Treatment of FN

Where FN occurs during a chemotherapy cycle, the Multinational Association of Supportive Care in Cancer (MASCC) risk index score will be assessed before initiating an empiric antibiotic regimen. Treatment of FN (if applicable) will be left to the discretion of the investigator. General information on the overall management of FN will be collected at each site.

### Sample size calculation

An accuracy of 2.25% (half length of the confidence interval) for the true FN rate is required. Assuming an overall FN rate of 15%, around 1000 patients will need to be recruited. With this sample size, at least 150 FN episodes should be documented (taking into account that a patient could develop more than one FN episode), which would allow around 10 covariates to be included in the risk model for predicting FN.

To derive a risk model, the development of FN will be used as a binary outcome without taking into account whether the patient developed several febrile episodes. Logistic regression will be used to model the probability of FN development.

Analyses of the data will be stratified by age (younger or older than 65 years) and will examine interactions between age, comorbidities, and other risk factors identified during development of the logistic regression model.

### Quality of life assessment

Assuming a mean overall Trial Outcome Index-Neutropenia Score of − 0.61 for patients with grade 3 to 4 neutropenia and an estimated 20% of patients expected to develop grade 3 to 4 neutropenia by day 8 of the first chemotherapy cycle, ~ 30 patients with grade 3 to 4 neutropenia should be recruited to detect this effect size (two-sided α = 5%, β = 10%); meaning ~ 150 patients with a QoL assessment should be recruited in total. Based on this calculation, 2 to 3 sites are required to participate in the QoL sub-study.

### Availability of data and materials

Not applicable.

## Discussion

This real-world study will include patients who are generally excluded from RCTs and analyses will be performed on data stratified by age (i.e. under and over 65 years of age) to provide a greater understanding of FN risk in elderly patients receiving myelosuppressive chemotherapy regimens with an expected moderate (10% to 20%) FN risk. The risk group of FN between 10 to 20% is very heterogeneous. This study will characterise the real risk of FN with the most common chemotherapy regimens used in clinical practice. It will also identify predictive factors associated with FN in this patient population and will develop a risk model assessment for this group of patients.

Clinicians frequently use data from RCTs to estimate the FN risk associated with a particular chemotherapy regimen [10, 14]; however, reported rates vary considerably for the same chemotherapy regimen. This variation may relate to differences in study populations; furthermore, the patient populations in these trials are often highly selected and may not be representative of the majority of patients treated in the general cancer population [10, 14]. It is also of note that guideline recommendations are generally based on results of RCTs and, as such, may not reflect daily clinical practice.

Evidence suggests that FN rates in the real-world setting may be higher than those reported in RCTs. In a systematic review and meta-analysis of 65 observational (*n* = 7812 patients) and 110 RCT (*n* = 42,257 patients) cohorts involving 29 breast cancer chemotherapy regimens, the unadjusted FN rate was 11.7% and 7.9% in the observational and RCT cohorts, respectively. FN rates remained significantly higher in the observational cohorts (*P* = 0.012) after adjusting for age, chemotherapy intent and regimen [14].

In the PACS 01 trial, which compared 6 cycles of fluorouracil, epirubicin and cyclophosphamide (FEC) with a sequential regimen of 3 cycles of FEC followed by 3 cycles of fluorouracil, epirubicin, and docetaxel (FEC-D) as adjuvant treatment for women with node-positive early breast cancer, the FN rate was 11.2% in the FEC-D arm, which did not meet the 20% risk threshold [15]; however, a meta-analysis of data from 9 studies conducted in routine clinical practice showed that patients treated with adjuvant FEC-D without G-CSF primary prophylaxis had an FN rate of 31%, exceeding the threshold [16].

Real-world data from patients aged < 65 years in Belgium showed the overall incidence of FN with chemotherapy regimens carrying a moderate or high risk of FN for the treatment of breast cancer and Hodgkin lymphoma was higher than expected, based on those reported in the literature from clinical trials [17].

Considering the available real-world evidence for the incidence of FN in patient populations undergoing chemotherapy with a FN risk of 10% to 20%, data are limited but a few observational studies report data from different patient populations.

A retrospective analysis was performed to determine the incidence of FN in 466 Japanese patients with non-Hodgkin lymphoma treated with R-CHOP [18]. Without G-CSF support, the incidence of FN was 9.1% in cycle 1 and 12.3% throughout all cycles. Of the patients with FN, 73.7% developed FN during cycle 1. Risk factors associated with the development of FN included albumin < 35 g/L, relative dose intensity < 85% and lack of G-CSF prophylaxis [18]. These findings suggest that, in this population, patients with these risk factors may benefit from G-CSF prophylaxis from cycle 1. Data from an observational study of 1829 European (97%) and Australian (3%) patients with non-Hodgkin lymphoma receiving CHOP (±rituximab) without G-CSF support showed that 18% of patients experienced at least one FN event [19]. Patients developing FN tended to be older (by about 4 years) and a higher percentage of them had advanced disease and a predicted risk of FN of ≥20% compared with matched controls.

The difference in FN incidence (12.3% vs. 18%) between these two non-Hodgkin lymphoma populations may be due to the ethnicity of the patients and is worthy of further investigation. Nonetheless, it should also be considered that not all patients in the European/Australian study received rituximab.

A retrospective study of 610 Korean women with early-stage breast cancer receiving adjuvant chemotherapy with AC (doxorubicin, 60 mg/m^2^ and cyclophosphamide, 600 mg/m^2^ every 21 days) reported FN in 8.5% of patients [20]. Predictors for FN included the presence of grade 4 neutropenia and a pre-treatment calculated estimated glomerular filtration rate less than 60 ml/min [20].

These real-world studies show differences in FN incidence between different patient populations and highlight that predictors of FN can be identified that may help advise on which patients may benefit most from G-CSF prophylaxis. Differences in FN between patients of different ethnicity is worthy of further investigation.

MONITOR-GCSF was an observational study in patients with solid or haematological malignancies receiving G-CSF support with Sandoz biosimilar filgrastim (EP2006/Zarzio^®^) for myelosuppressive chemotherapy [21]. Data from the study were used in a predictive model to identify determinants of febrile neutropenia episodes [21]. Patients were more likely to experience an FN episode if they had Eastern Cooperative Oncology Group status ≥2 anytime during the study or received antibiotic prophylaxis. Notably, patients who were ‘under-prophylacted’ with G-CSF were also more likely to experience FN [22]. Rates of FN were consistently lower in over-prophylacted patients compared to those under- and correctly-prophylacted. Under-prophylacted patients were at higher risk for disturbances to their chemotherapy regimens [23].

Stratified analyses performed in elderly patients showed that the only predictive factor of an elderly patient experiencing an FN episode was receiving antibiotic prophylaxis [24].

Evidence suggests that the availability of G-CSF biosimilars has led to cost savings, compared with use of the reference product [25, 26]; indeed, since 2015, filgrastim has been included on the World Health Organisation (WHO) Model List of Essential Medicines, reflecting its cost-effectiveness [27]. Since cost-savings associated with biosimilars has implications for increasing access to G-CSF supportive care, it is important to characterise the subset of patients at 10% to 20% risk of neutropenia who are most likely to benefit from G-CSF prophylaxis. This is particularly of note since the 10% to 20% subset of patients are a very heterogeneous population.

Our study can build on the predictors identified in observational studies including MONITOR-GCSF and provide information on which patients with a 10% to 20% risk of neutropenia will be most likely to benefit from G-CSF prophylaxis. Since the study will include patients generally not included in clinical trials, the findings will likely differ to published findings from RCTs and may be practice-changing.

### Trial status

The study began recruiting patients in December 2016. Recruitment is expected to be completed by 31 December 2018. Total study duration is estimated to be 36 months. The study objective and design was introduced at the 2017 MASCC Annual Meeting in Washington DC, USA (June 22─24 2017) by the MASCC study group Chair (Jean Klastersky) and Vice-Chair (Bernardo Rapoport). Please visit http://www.mascc.org/neutropenia-infection-myelosuppression for further details.

## Abbreviations

FEC: fluorouracil, epirubicin and cyclophosphamide
FEC-D: fluorouracil, epirubicin and docetaxel
FN: febrile neutropenia
G-CSFs: granulocyte colony-stimulating factors
MASCC: Multinational Association of Supportive Care in Cancer
QoL: quality of life
RCTs: randomised controlled trials
